# Morphology and morphometry of the caudate lobe of the liver in two populations

**DOI:** 10.1007/s12565-016-0365-7

**Published:** 2016-09-01

**Authors:** Mandeep Gill Sagoo, R. Claire Aland, Edward Gosden

**Affiliations:** 1 Biomedical Sciences, King’s College, London, London, United Kingdom; 2 School of Rural Medicine, University of New England, Armidale, Australia; 3 Research Methods Group, Institute of Health and Biomedical Innovation, Queensland University of Technology, Brisbane, Australia; 4 Government Medical College, Amritsar, India; 5 St George’s, University of London, London, United Kingdom

**Keywords:** Caudate lobe, Caudate process, Cirrhosis, Hepatic index, Liver, Papillary process

## Abstract

The caudate lobe of the liver has portal blood supply and hepatic vein drainage independent of the remainder of the liver and may be differentially affected in liver pathologies. Ultrasonographic measurement of the caudate lobe can be used to generate hepatic indices that may indicate cirrhosis. This study investigated the relationship of metrics of the caudate lobe and other morphological features of human livers from a northwest Indian Punjabi population (*n* = 50) and a UK Caucasian population (*n* = 25), which may affect the calculation of hepatic indices. The width of the right lobe of the liver was significantly smaller, while the anteroposterior diameter of the caudate lobe and both Harbin’s Index and the Hess Index scores were significantly larger in NWI livers than in UKC livers. The Hess Index score, in particular, is much larger in the NWI population (265 %, *p* < 0.005). Two caudate lobe features were significantly different between the two populations—the shape of the caudate lobe and the development of the caudate process. This study shows significant population differences exist in several metrics and morphological features of the liver. These differences may affect the calculation of hepatic indices, resulting in a greater percentage of false positives of cirrhosis in the NWI population. Population-specific data are required to correctly determine normal ranges.

## Introduction

The caudate lobe is one of four anatomical lobes of the liver. The lobe is bounded on the left by the fissure for the ligamentum venosum, inferiorly by the porta hepatis and on the right by the groove for the inferior vena cava. Superiorly, it continues into the superior surface of the right upper end of the fissure for the ligamentum venosum (Dodds et al. 1990).

The caudate lobe is subdivided into Spiegel’s lobe (the caudate lobe proper and the papillary process), the caudate process and the paracaval portion, which is anterior to the inferior vena cava (Murakami and Hata 2002). The caudate lobe is connected to the right lobe of the liver by the caudate process, which passes laterally between the portal vein and the inferior vena cava at the porta hepatis. The caudate process is sometimes elevated. The medial inferior part of the caudate lobe sometimes forms a papillary process, which passes left (and also sometimes anteriorly) into the region of the superior recess of the omental bursa. The caudate and papillary processes are sometimes separated from the remainder of the liver by grooves or fissures (Auh et al. 1984).

Functional divisions of the adult liver are defined by vascular organisation; several classifications are currently used. The Glissonian system divides the liver into two based on the bifurcation of the portal vein into the left and right branches (Cantlie 1898, for example). Couinaud (1957) divided the adult liver using the distribution of the portal venous system as follows: The portal vein divides into the left and right branches, both of which divide again, producing four main branches, which each supply a portal sector (Goldsmith and Woodburne 1957). Each portal sector is further subdivided by the portal vein. The eight resulting segments are numbered I–VIII clockwise from the inferior vena cava (IVC). Segment I in Couinaud’s classification is the caudate lobe, which, alone of the segments, receives blood independently from both the left and right portal veins (Murakami and Hata 2002).

Systemic arterial supply to the caudate lobe is usually derived from multiple caudate arteries, which arise from the right, left and middle (if present) hepatic arteries. These arteries are frequently connected to each other and have overlapping vascular territories (Mizumoto and Suzuki 1998).

Most of the liver drains into the inferior vena cava by three major hepatic veins, left, right and middle. The caudate lobe, however, drains via several minor hepatic veins (between 1 and 5), which open into the inferior vena cava independently of the major hepatic veins (Filipponi et al. 2000; Sagoo and Agnihotri 2009). The caudate lobe is therefore anatomically and functionally independent of the right and left lobes of the liver (Abdalla et al. 2002).

The differential blood supply and drainage by the portal and hepatic veins suggest a mechanism by which the caudate lobe might be affected differentially from the remainder of the liver by vascular-based pathologies. In Budd-Chiari syndrome in which hepatic venous outflow is blocked, the caudate lobe often continues to drain unimpeded and undergoes a compensatory hypertrophy (Filipponi et al. 2000). The caudate lobe may also be spared from the hepatic parenchymal atrophy of cirrhosis and undergo compensatory hypertrophy (Mullane and Gliedman 1966).

The differential effect of cirrhosis on the lobes of the liver has been suggested as the basis for ultrasonographic diagnosis of cirrhosis. In cirrhotic livers, the right lobe exhibits relatively greater shrinkage, while the caudate lobe undergoes relative enlargement (Harbin et al. 1980). Using the ratio of transverse caudate lobe width to transverse right lobe width—the Harbin Index—cirrhotic livers could be separated from non-cirrhotic livers (both healthy and those with other hepatic pathologies) with a sensitivity of 84 % and a specificity of 100 %. Several other hepatic indices (Hess Index, Porta Hepatis Index) have been proposed (Harbin et al. 1980; Hess et al. 1989) in an attempt to compare caudate lobe volume with the volume of the remainder of the liver before decisions about further, potentially invasive investigations (Giorgio et al. 1986).

The use of ratios as opposed to absolute measures allows description of differential changes within the liver taking into account changes in overall liver volume and thus eliminating the need for body size corrections. However, because the measurements used in ratios are taken from defined points on the liver, variations in the morphology of the liver may influence the metrics of the caudate lobe and any indices calculated from them.

The shape of the caudate lobe proper is also variable, as are the shapes of its anterior and upper margins (Sahni et al. 2000). Pons hepaticus, caudate and papillary processes are also variably present and developed morphological features of the caudate lobe (Chang et al. 1989; Sahni et al. 2000).

It is not known whether population differences exist in overall liver size or caudate lobe size or whether the presence and development of morphological features affect indices.

This present study compared two populations—southeast UK Caucasian and northwest Indian—to investigate population differences in caudate lobe morphology and morphometry. Previously published techniques for measuring the caudate lobe, the hepatic indices, were used as measures of caudate lobe size.

## Materials and methods

The study was conducted according to the ethical and legal standards and approval of the respective institutions and countries. Copies of the approvals have been provided to the journal and are available from the corresponding author.

### Specimens

The study was conducted on 50 northwest Indian (NWI) livers obtained from the Department of Anatomy, Government Medical College, Amritsar, India, and 25 Caucasian (UKC) livers from St George’s, University of London, UK. These livers were from donors to the respective anatomy programmes at each institution. Cadavers were embalmed, with livers in situ, using formalin-based embalming fluid. Ages ranged between 50 and 95 years. Gender information was not collected as part of this study.

Any livers from cadavers with previous history or appearance of cirrhosis, metastatic disease or other liver pathology and any cadavers with previous history of or an appearance suggestive of abdominal surgery, trauma or disease were excluded from the study.

### Hepatic measurements and indices calculations

All hepatic measurements were performed on intact livers. Digital vernier calipers and cotton threads were used to measure hepatic parameters; all measurements were taken by the same person, the first author.

Data are summarised in Table 1.

**Table 1:** Definitions of hepatic measurements

Hepatic measurement	*C*_T_—transverse diameter of the caudate lobe^a^	*C*_L_—longitudinal diameter of the caudate lobe	*C*_AP_—antero-posterior diameter of the caudate lobe	SD—sagittal diameter of the porta hepatis	TD—transverse diameter of the porta hepatis	RL—right lobe diameter^b^
Description	Measured from the most medial margin of the caudate lobe to the right lateral wall of the portal vein	Measured at the level of the greatest longitudinal extension of the caudate lobe	Measured at the level of the greatest anterolateral extension of the caudate lobe	The greatest sagittal diameter of the porta hepatis measured with the axis of measurement passing through the main portal vein, just before bifurcation	The greatest transverse diameter of the porta hepatis measured with the axis of measurement passing through the main portal vein just before bifurcation. The most medial aspect of the caudate lobe was used as the medial extent of the transverse diameter of the porta hepatis	From right lateral wall of the portal vein to the most lateral margin of the right lobe

^a^Defined as measurement A by Harbin et al. 1980

^b^Defined as measurement X by Harbin et al. 1980

**Fig. 1:**
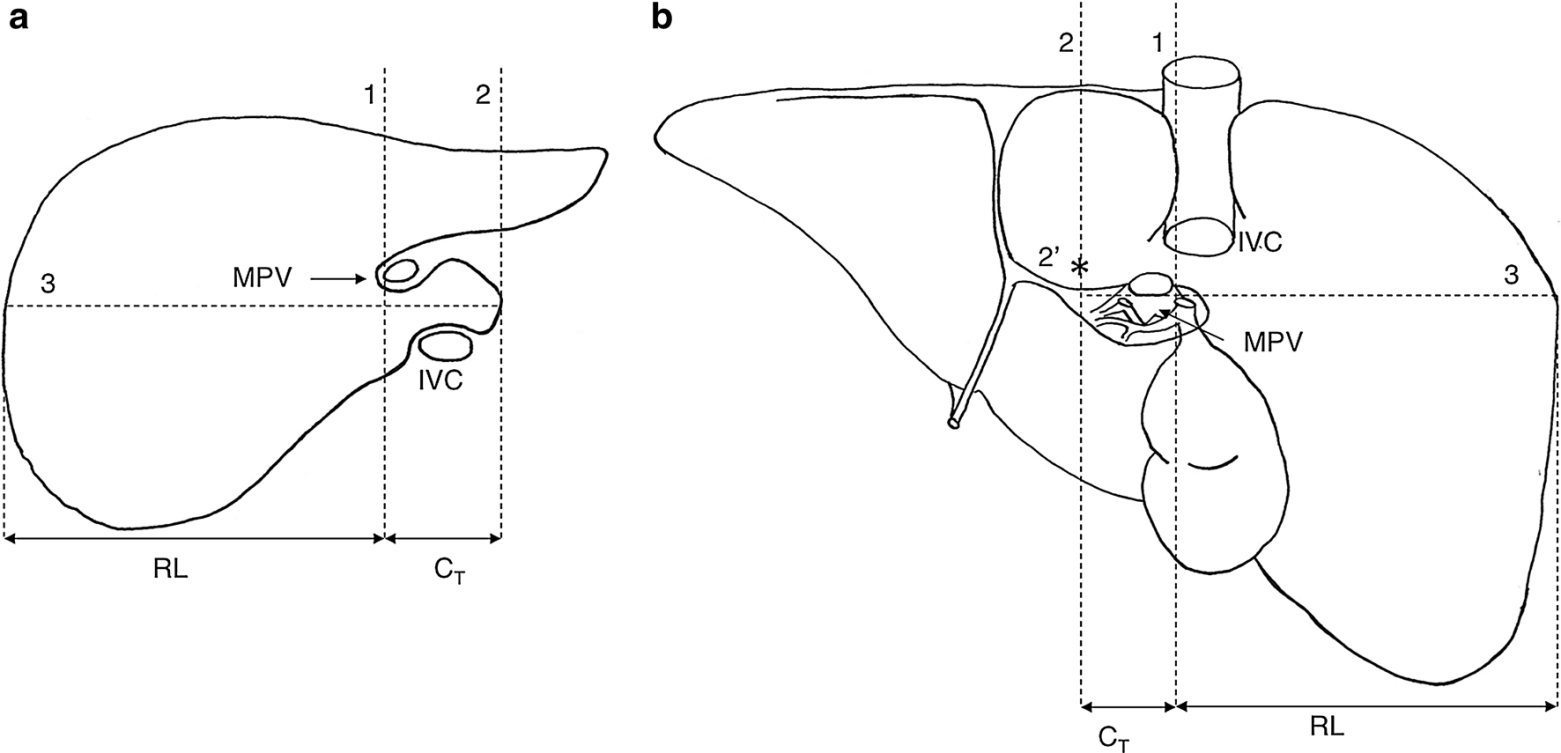
Schematic drawings of liver, showing landmarks for caudate to right lobe ratio (*C*_T_/RL). **a** Transverse section of liver, adapted from Harbin et al. (1980) Fig. 1, showing their original landmarks, drawn through the porta hepatis, at the branching of right portal vein from the main portal vein. **b** Visceral surface of liver, showing the adapted landmarks used in the present study. The *first line* (1) is drawn at the level of the right lateral margin of the main portal vein (MPV) parallel to the midsagittal plane. A *second line* (2) is drawn at the level of the most medial part of the caudate lobe. The *asterisk* labelled 2′ indicates the most medial aspect of the caudate lobe, through which line 2 was drawn. A *third line* (3) is drawn perpendicular to the first two lines, to the most lateral margin of the right lobe. The diameter of the right lobe (RL) and the transverse diameter of the caudate lobe (*C*_T_) are measured. *IVC* inferior vena cava

**Fig. 2:**
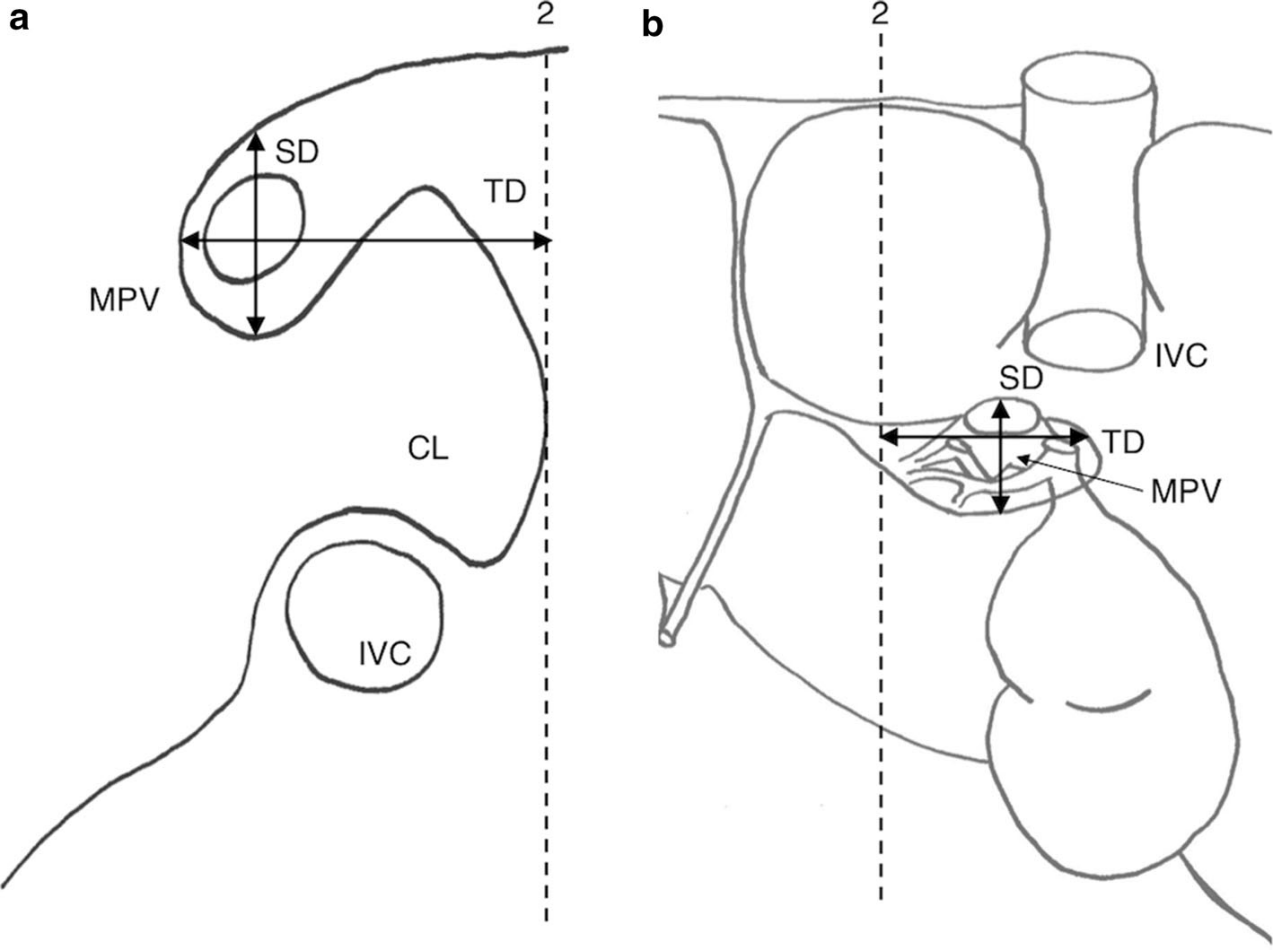
Schematic drawing of liver, showing additional landmarks for calculation of the Porta Hepatis Index (SD × TD)/RL. **a** Transverse section through porta hepatis, at branching of right portal vein from the main portal vein. **b** Visceral surface of liver, showing the adapted landmarks used in the present study. The transverse diameter of the porta hepatis (TD) is measured from a line drawn from the lateral extent of the porta hepatis to a line drawn through the most medial aspect of the CL (2), passing through the MPV. The sagittal diameter of the porta hepatis (SD) is drawn at maximum anteroposterior extent of the porta hepatis. The axis of measurement for both SD and TD pass through the MPV (adapted from Harbin et al. (1980) (**a**, **b**)

**Fig. 3:**
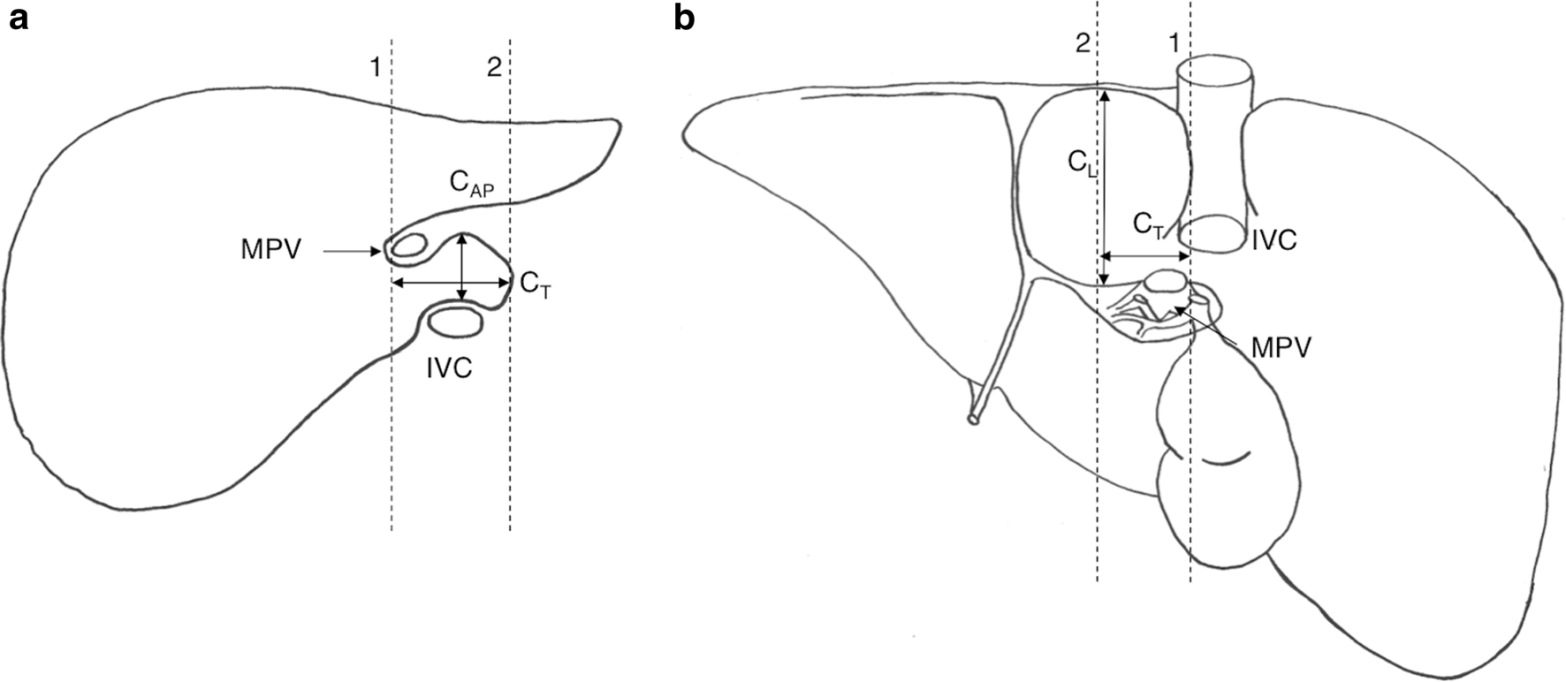
Schematic drawings of liver, showing additional landmarks for calculation of the Hess Index (*C*_L_ × *C*_AP_ × *C*_T_)/RL. **a** Transverse section of liver, adapted from Harbin et al. (1980) Fig. 1, demonstrating the greatest anteroposterior diameter of the caudate lobe (*C*_AP_) measured parasagittally through the porta hepatis at the part of caudate lobe that forms the roof of the porta hepatis. **b** Visceral surface of the liver, demonstrating the greatest longitudinal diameter of the caudate lobe (*C*_L_) measured supero-inferiorly. *C*_T_ transverse diameter of the caudate lobe, *IVC* inferior vena cava, *MPV* main portal vein

1. Harbin’s Index *C*_T_/RL
  This ratio was calculated by drawing a line (line 1) through the right lateral wall of the main portal vein (MPV). Line 2 was drawn parallel to line 1 at the most medial aspect of the caudate lobe. Line 3 was drawn perpendicular to lines 1 and 2, midway between MPV and the inferior vena cava (IVC), and extended out to the lateral margin of the right lobe. The distance along line 3, between lines 1 and 2, was the transverse diameter of the caudate lobe (*C*_T_) and corresponded to Harbin’s measurement A; the distance along line 3, between the lateral margin of the right lobe and line 1, was the right lobe diameter (RL) and corresponds to Harbin’s measurement X. Figure 1 illustrates these landmarks on a typical intact liver.

(2) Porta Hepatis Index (SD × TD)/RL
  The Porta Hepatis Index score was calculated as per Harbin’s original description: The transverse (TD) and sagittal diameters (SD) of the porta hepatis were measured with the axis of measurement crossing the main portal vein just prior to bifurcation. In Harbin’s original study, two approaches were taken for defining TD; the greatest transverse diameter was used where there was a clear-cut porta hepatis and the most medial margin of caudate lobe was used for a less-defined porta hepatis. For consistency, the present study used the most medial aspect of the caudate lobe as the medial extent of the transverse diameter of the porta hepatis (TD) in all livers. The sagittal diameter (SD) was measured perpendicular to the TD, with the axis of measurement passing through the MPV. Figure 2 illustrates these measurements.

(3) Hess Index (*C*_L_*C_AP_*C_T_)/RL
  These measurements were easily located on a liver that was removed intact and required no alteration from Hess’ original descriptions. Figure 3 describes these measurements.

### Shape of the caudate lobe

The overall shape of the caudate lobe was classified into rectangular, piriform or irregular (Table 2).

**Table 2:** Classification criteria for caudate lobe shape (definitions follow Sahni et al. 2000)

Shape	Piriform	Rectangular	Irregular
Classification criteria	Caudate lobe wider at either superior or inferior extent, narrower at the opposing extent	Caudate lobe roughly equal size at both superior and inferior extents, overall presents a rectangular shape	Caudate lobe has irregular boundaries and does not fit either piriform or rectangular classifications

The shapes of the anterior and upper margins of the caudate lobe were classified into either convex, concave, concavo-convex or irregular (as per Sahni et al. 2000). The presence (complete or partial) or absence of a pons hepaticus and the development of caudate and papillary processes were recorded.

The pons hepaticus was defined as complete when it completely covered the posterior aspect of the retrohepatic segment of inferior vena cava. It was defined as partially complete when it partially covered the posterior aspect of the retrohepatic segment of inferior vena cava.

A papillary process was considered to be present when it formed a prominent elevation and/or was separated from the caudate lobe proper by a partial or complete fissure and separated by the groove for the ligamentum venosum from the left lobe of the liver and the porta hepatis inferiorly.

The caudate process was considered well developed when it formed a prominent elevation and/or was separated from the caudate lobe proper by a partial or complete fissure, extending towards the right lobe and almost forming a relatively wide roof over the porta hepatis.

### Statistical analysis

All statistical analyses and tabulations were performed using IBM SPSS Statistics for Mac, version 22.0 (Armonk, NY: IBM Corp.).

Descriptive statistics: mean, standard deviation, median, minimum, maximum, skewness and kurtosis were calculated for each measure recorded. Parameters that were considered to have a normal distribution (skewness and kurtosis in the range >−3 and <+3) were compared using independent samples *t* tests; the others were compared using Mann-Whitney *U* tests.

The number and percentage of each morphological feature were recorded for livers in both populations. Population differences in morphology were analysed using Pearson’s chi square or Fisher's exact test, where cell counts were low (2 or less); in some cases, morphological categories were combined to produce adequate cell counts, where this was considered appropriate.

## Results

### Hepatic measurements and indices

A summary of the hepatic measurements and indices for both populations is presented in Tables 3, 4 and 5.

**Table 3:** Hepatic measurements and Harbin Index: mean and standard deviation (cm), median (cm) (minimum and maximum values), skewness and kurtosis

Parameter	*C*_T_				RL				Harbin Index (*C*_T_/RL)			
Statistic	Mean (SD)	Median (min, max)	Skewness	Kurtosis	Mean (SD)	Median (min, max)	Skewness	Kurtosis	Mean (SD)	Median (min, max)	Skewness	Kurtosis
UKC	2.36 (0.75)	2.20 (1.4, 4.7)	1.27	2.45	8.82 (1.09)	8.60 (7.0, 11.4)	0.48	0.24	0.27 (0.1)	0.26 (0.16, 0.6)	1.37	1.65
NWI	2.74 (1.22)	2.50 (1.0, 7.5)	1.72	4.10	8.06 (1.06)	8.00 (5.0, 10.6)	−0.14	0.77	0.343 (0.15)	0.29 (0.15, 0.83)	3.38	3.00

**Table 4:** Hepatic measurements and Porta Hepatis Index: mean and standard deviation (cm), median (cm) (minimum and maximum values), skewness and kurtosis

Parameter	SD				TD				Porta Hepatis Index [(SD × TD)/RL]			
Statistic	Mean (SD)	Median (min, max)	Skewness	Kurtosis	Mean (SD)	Median (min, max)	Skewness	Kurtosis	Mean (SD)	Median (min, max)	Skewness	Kurtosis
UKC	2.38 (0.52)	2.30 (1.20, 3.40)	−0.37	0.15	2.14 (0.64)	3.10 (1.40, 4.00)	−0.409	−0.237	0.80 (0.267)	0.8 (0.30, 1.43)	0.21	0.13
NWI	2.13 (0.70)	2.30 (0.20, 4.10)	0.37	1.31	2.74 (0.72)	2.60 (1.3, 5.0)	0.55	1.05	0.743 (0.334)	0.68 (0.04, 1.71)	1.00	1.39

**Table 5:** Hepatic measurements and Hess Index: mean and standard deviation (cm), median (cm) (minimum and maximum values), skewness and kurtosis

Parameter	*C*_L_				*C*_AP_				Hess Index [(*C*_L_ × *C*_AP_ × *C*_T_)/RL]			
Statistic	Mean (SD)	Median (min, max)	Skewness	Kurtosis	Mean (SD)	Median (min, max)	Skewness	Kurtosis	Mean (SD)	Median (min, max)	Skewness	Kurtosis
UKC	5.44 (1.24)	5.80 (2.6, 7.2)	−0.43	−0.66	1.65 (0.80)	1.50 (0.13, 3.0)	0.14	0.10	2.66 (2.07)	2.16 (0.09, 9.75)	1.76	4.67
NWI	5.74 (1.41)	6.0 (3.0, 8.2)	−0.15	−0.97	4.36 (0.77)	4.25 (2.5, 6.3)	0.14	0.10	8.2 (3.23)	7.89 (2.33, 16.2)	0.50	−0.36

CT and RL measures are in Table 3a

The *C*_T_, Harbin Index and Hess Index all displayed kurtosis in excess of 3 (Tables 3, 5) and therefore were not considered sufficiently normally distributed to analyse using t tests. Levene’s test for homogeneity of variance (UK vs. Indian population) was insignificant for all parameters that were analysed by *t* tests (*P* > 0.05), so there was considered to be no requirement to correct for unequal variance. Median and range for the Hess Index are both greater for the NWI population than the UKC.

Mean differences in the C_T_, Harbin Index and Hess Index were compared using non-parametric tests (Mann-Whitney test) (Table 6), and differences in all other parameters were compared using independent samples t tests (Table 7). In all cases, two-sided tests and a significance level of 0.05 were used. The UKC livers were designated the reference group, so, in Tables 6 and 7, a positive value means that the metric was larger in the NWI livers than in the UK livers and a negative one that it was smaller. Parameters that showed significant differences (*p* < 0.05) between UKC and NWI groups were Harbin’s Index, Hess Index, RL and *C*_AP_.

**Table 6:** Comparisons between UKC and NWI livers (Mann-Whitney tests)

Parameter	*C*_T_	Harbin’s Index	Hess Index
Difference between medians^a^	0.30	0.04	5.73
Percentage difference^a^	13.6	13.6	265
*U*	519	447	71
*p*	0.23	0.05	<0.005

^a^NWI population in relation to UKC population

**Table 7:** Comparisons between UKC and NWI livers (independent samples *t* tests)

Parameter	RL	SD	TD	Porta Hepatis Index	*C*_L_	*C*_AP_
Difference between means^a^	−0.76	−0.25	0.60	−0.11	0.2	2.71
Percentage difference^a^	−8.7	−10.6	−16.2	−7.4	3	164
*t*	2.92	1.54	1.17	0.79	−0.91	−13.96
*p*	0.005	0.13	0.25	0.43	0.37	<0.005

^a^NWI population in relation to UKC population

The ranges originally defined as normal or cirrhotic for Harbin, Porta Hepatis and Hess Indices (Harbin et al. 1980; Hess et al. 1989) were applied to the livers in this study. Three NWI livers (3/50) were classified as cirrhotic by the Harbin Index, while all of the UKC livers were classed as non-cirrhotic. Three UKC livers fell into the cirrhotic group (3/25) using the Hess Index, whilst 39 of the NWI livers (39/50) were classed as cirrhotic and only 11 as non-cirrhotic.

A two-step cluster analysis with automatic determination of the number of clusters was performed on the data, which resulted in two clusters corresponding exactly to the NWI and UKC populations. There was no evidence of any additional clusters that might correspond to healthy or diseased in either population.

A logistic regression of population onto *C*_AP_ demonstrates that *C*_AP_ alone could predict population of origin with 97 % accuracy in this data set. That is, all but two individuals from 75 can be correctly classified into population of origin using just this metric.

### Morphological features of the caudate lobe

**Fig. 4:**
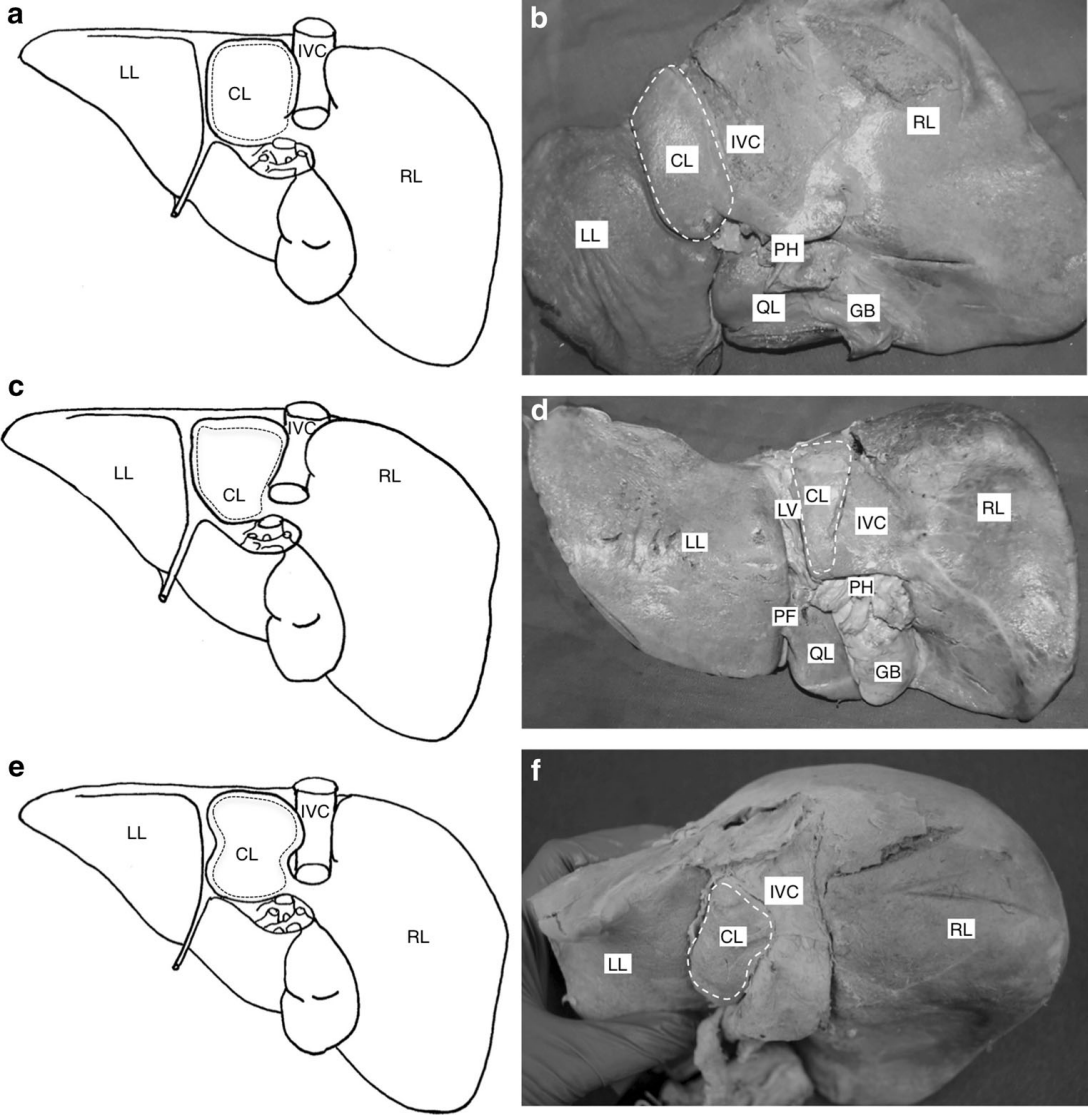
Schematic drawings and representative photographs demonstrating various caudate lobe shapes. *Dotted lines* emphasise the shape of the caudate lobe. **a**, **b** Visceral surface of liver, diagram and photograph of rectangular caudate lobe. **c**, **d** Visceral surface of liver, diagram and photograph of piriform caudate lobe. **e**, **f** Visceral surface of liver, diagram and photograph of irregular caudate lobe. *CL* caudate lobe, *GB* gallbladder, *IVC* inferior vena cava, *LL* left lobe, *RL* right lobe, *PH* porta hepatis, *QL* quadrate lobe

Examples of rectangular, piriform and irregular shaped caudate lobes were seen in both populations, as were examples of complete, partial and absent pons hepaticus, papillary processes and well-developed caudate processes. The caudate lobe shapes are illustrated in Fig. 4.

The prevalence of features of the caudate lobe in both populations are summarised in Table 8.

**Table 8:** Prevalence of morphological features of the caudate lobe in NWI and UKC livers

Feature	Caudate lobe shape			Pons hepaticus			Caudate process	Papillary process	Caudate lobe anterior margin				Caudate lobe upper margin		
	Rectangular	Piriform	Irregular	Complete	Partial	Absent	Well-developed	Present	Convex	Concave	Concavo-convex	Irregular	Convex	Concave	Concavo-convex
NWI *n* (%) (*n* = 50)	45 (90 %)	3 (6 %)	2 (4 %)	1 (2 %)	12 (24 %)	37 (74 %)	30 (60 %)	17 (34 %)	43 (86 %)	4 (8 %)	1 (2 %)	2 (4 %)	40 (80 %)	0 (0 %)	10 (20 %)
UKC *n* (%) (*n* = 25)	4 (16 %)	8 (32 %)	13 (52 %)	3 (12 %)	7 (28 %)	15 (60 %)	22 (88 %)	13 (52 %)	20 (80 %)	3 (12 %)	2 (8 %)	0 (0 %)	20 (80 %)	0 (0 %)	4 (16 %)

A summary of the analysis of differences in morphological features between the two populations is presented in Table 9.

**Table 9:** Analysis of population differences in morphology

Feature	Caudate shape^a^	Presence of caudate process	Presence of papillary process	Pons hepaticus	Anterior margin of caudate lobe	Upper margin of the caudate lobe^b^
Test statistics	Pearson’s chi square	Pearson’s chi square	Pearson’s chi square	Fisher’s exact test	Fisher’s exact test	Pearson’s chi Square
Result	40.3	6.15	2.25	3.5	0.78	0.0
*df*	1	1	1		2	2
*p* value	<0.005	0.01	0.13	0.16	0.69	1.0

^a^Irregular and piriform were combined into one category

^b^Irregular and concavo-convex were combined into one category

The majority of NWI caudate lobes were rectangular, whereas UKC ones were irregular; this difference was highly significant (*p* ≤ 0.001). The caudate process was significantly more common in the UKC population than in the NWI population (*p* = 0.013). However, there was no significant difference in the presence of the papillary process or the presence (complete or partial) of the pons hepaticus. There were no significant differences in the shapes of the anterior and upper margins of the caudate lobe, which, in both populations, were predominantly convex.

## Discussion

This study has demonstrated that several metrics and morphological features in healthy livers differ significantly between NWI and UKC populations and that these differences, in turn, may affect calculation of hepatic indices that are used to indicate cirrhosis. Four parameters differed significantly between the two populations: two of these were simple metrics, the mean width of the right lobe of the liver (RL) and the mean anteroposterior diameter of the caudate lobe (*C*_AP_). The others were ratios calculated using these metrics—the Harbin and Hess Indices. In the NWI population, RL was slightly smaller (8.7 %) than the UKC population, which most likely resulted in the significantly higher (13.6 %, *p* = 0.045) Harbin Index in the NWI population as right lobe width forms the denominator of this calculation. However, the mean *C*_AP_ is very much larger (164 %) in the NWI population, which results in the median Hess Index being much larger in that population (265 %, *p* < 0.001) than in the UKC population. Whilst differing simple metrics may simply indicate that the overall size of the livers differs between the two populations, the differing ratios calculated from them demonstrates that there are significant differences in the relative sizes of parts of the liver.

Higher Harbin and Hess Index scores in NWI livers could possibly increase the number of false positives if these indices are used to investigate cirrhosis. Application of the original cut-off for the Hess Index (derived from a UKC population) to an NWI population would result in 78 % of the NWI livers falling into the ‘cirrhotic’ group, which is an improbable result since the prevalence of hepatitis B and C infections in the general population of the Punjab is 2.3 and 3.0 %, respectively (Alia et al. 2009), which also could not explain a 78 % prevalence of undiagnosed cirrhosis in this population of cadavers.

The hepatic indices were originally calculated from measures obtained using ultrasound in living individuals, with a subsequent histological confirmation of cirrhosis. It was possible to identify the same points to take measurements on embalmed livers as described in the original papers by Harbin and Hess, but histological confirmation of absence of hepatic disease was not possible in this study. However, there was no macroscopic evidence of hepatic disease in any liver included in this study and a cluster analysis sorted into two clusters only, corresponding exactly to the original population groupings, and did not indicate the existence of clusters that could be interpreted as ‘diseased’; therefore it is considered that the differences between the UKC and NWI populations are true population differences rather than indicative of a difference in the prevalence of cirrhosis in the cadaver populations.

Measures of body size were not taken in this study, nor in other studies on hepatic indices, so it is not possible to directly investigate whether there is a differential effect of body size on segments of the liver. However, the association between body size and total liver volume has been investigated (DeLand and North 1968; Urata et al. 1995; Heinemann et al. 1999; Vauthey et al. 2002). Vauthey et al. (2002) found in their CT study that body surface area and body weight were predictors of total healthy liver volume in Western adults. Similar predictors of total liver volume in relation to body size have been calculated in a Japanese population (Urata et al. 1995) and using autopsy-derived data from Western populations (DeLand and North 1968; Heinemann et al. 1999). Application of either the Japanese formulae or the autopsy-derived formulae to Vauthey and colleagues’ (2002) CT data resulted in under- or over-estimation of total liver volume, respectively, indicating that both population of origin and technique used may influence the relationship. Again, the cluster analysis performed showed only the two original populations and did not suggest subgroupings that could identified as sex.

A point that should be considered is whether measurements developed using ultrasound on a living patient can be applied to fixed tissue without modification. It is known that tissues shrink following immersion fixation in formalin (>10 % in liver, Rutherford and Karanjia 2004) but it is unknown what shrinkage may occur in embalmed cadavers. It is assumed that similar behaviour occurred in both populations examined in the present study, as they were embalmed in similar manners. The homogeneity of healthy liver tissue implies that an assumption of uniform shrinkage in all dimensions is probably justified.

Two morphological features—overall shape of the lobe and the presence of a well-developed caudate process—differed significantly between the two populations. The results of this study are in agreement with other reports that found the majority of adult NWI livers had rectangular-shaped caudate lobes (94.5 %, Sahni et al. 2000; 58 %, Joshi et al. 2009). In the UKC population in the present study, the least common caudate lobe shape was rectangular. Well-developed caudate processes are present in a majority of livers, in both the NWI (60 %—present study, 59.5 %—Sahni et al. 2000) and UKC livers (80 %—present study). The difference in prevalence between the two populations, however, is statistically significant. Caudate process variability has also been reported in a North American study (Auh et al. 1983). It is unlikely that the differing prevalence of caudate lobe shape and caudate process development could affect either RL or C_AP_, the two metrics that also differed significantly, as neither metric is measured in relation to these morphological features.

Caudate and papillary processes need to be considered in measures of caudate lobe volume. The caudate and papillary processes share the features of blood supply and drainage hypothesised to underlie the compensatory hypertrophy of the caudate lobe in cirrhosis (Ortale and Borges Keiralla 2004) and would presumably be affected as the caudate lobe itself is in hepatic disease. Hepatic indices do not include measures for caudate and papillary processes, which is a limitation of using them as estimators of relative size.

This study has demonstrated that several metrics and morphological features in healthy livers differ significantly between NWI and UKC populations. These differences may affect the calculation of the hepatic indices that are used to indicate cirrhosis and may result in increased false positives in the NWI population. Given the effect of *C*_AP_ on the Hess Index, it is suggested that, for this index to be used correctly, population-specific data on healthy individuals should be obtained and the normal range for healthy livers calculated. We also suggest that further studies be performed on the variability of morphological features of the caudate lobe and the effect that these may have on the calculations of caudate lobe volume.
